# Supplementary material for: A quasi-experimental effectiveness evaluation of the ’Incredible Years Toddler’ parenting programme on children’s development aged 5: A study protocol
Source: PLoS One. 2023 Sep 27;18(9):e0291557. doi: 10.1371/journal.pone.0291557 (PMC10529533; doi:10.1371/journal.pone.0291557)
Supplement: S2 File — (DOCX) [file pone.0291557.s002.docx]

Logic Model: Incredible Years Toddler Basic

## Need Inputs Activities Outputs Outcomes IMPACT

Parents report an improvement in:

- (Child) social and emotional development
- (Parent) understanding and confidence in using positive parenting strategies
- (Parent) mood or depression levels
- (Child) eating/food behaviours, physical activity, and screen time

Parents report satisfaction with the programme

N.B. For parents who started IY without difficulties we expect to see no deterioration in the outcomes above

**Short Term**:

More toddlers have good social and emotional development with lower levels of aggression in pre-schoolers.

Children at the age of 2 years have age appropriate development in communication and language skills and can use language to express their physical and emotional needs

**Medium term**:

More children will enter school with the language and communication skills they need to engage in this stage of early learning and to develop effective relationships.

**Long Term:**

Children in Primary school have improved school behaviour and engage positively with their peers and teachers.

More children will have better literacy and language skills through primary school.

Children will have better literacy and language skills and better achievement on leaving primary school.

No. of telephone contacts

No. of home visits

No. of referrals

No. of participants enrolled on programme

No. of parent courses delivered

- Virtual
- In person*

No. of course sessions participants attend

- Virtual
- In person*

No. of participants completing pre and post measures

No. of participants completing the programme

No. of supervision sessions staff attend

No. of accredited staff

Group facilitator Training

Data entry training

Engagement/referral activities

3 promotional contacts prior to starting on programme (assertive outreach)

1 follow up session to review and collect measures

- Telephone contact
- 2 x home visits*
- Zoom sessions

Introduction week

16 x 13week (2 – 2.5 hr) Programmes per year (160 participants)

8 parts of programme delivered in sequence (see Service Design document for details)

Pre and post measure data collection

Data entry by facilitators

Data monitoring

Celebration week

Ongoing staff supervision and Coaching

Project manager/Coordinator (1 x FTE)

Data administrator (1 x FTE)

Group Leader (3 x 22hrs, 3 x 30 hrs)

Group leader training & certification

4 facilitators to support provision of creche & support for families

Marketing & promotion

Programme materials

Equipment costs

IT Equipment

Zoom license

Crèche provision*

Venue hire*

Universal intervention for families with varying levels of need in relation to:

Parent concerns regarding the social & emotional development of their children aged 12-36 months

Parent lack knowledge, skills and/or positive parenting techniques

Parent low mood or depression

Other risk factors leading to poor quality parent infant relationships
